# Metagenomics-based characterization of fecal microbiome and resistome of laying hens during the production cycle

**DOI:** 10.3389/fvets.2025.1740567

**Published:** 2026-01-13

**Authors:** Ying-Qian Gao, Qing-Yu Hou, Xin-Wen Hou, Yong-Jie Wei, Kai-Meng Shang, He Ma, Hong-Li Geng, Rui Liu, Li-Hua Yang, Hany M. Elsheikha, Hong-Bo Ni, Yu-Feng Huang

**Affiliations:** 1 College of Veterinary Medicine, Qingdao Agricultural University, Qingdao, China; 2 College of Life Sciences, Changchun Sci-Tech University, Shuangyang, China; 3 Faculty of Medicine and Health Sciences, School of Veterinary Medicine and Science, University of Nottingham, Sutton Bonington Campus, Loughborough, United Kingdom

**Keywords:** antimicrobial resistance genes, fecal microbiota, laying hens, metagenomics, mobile genetic elements

## Abstract

The extensive use of antimicrobials in livestock has accelerated the emergence of antimicrobial resistance (AMR), raising serious global concerns. Poultry feces are recognized as important reservoirs of antibiotic resistance genes (ARGs) and their associated mobile genetic elements (MGEs); however, the microbial community characteristics and ARG profiles of laying hens across different laying stages remain poorly understood. In this study, 40 fecal samples were collected from laying hens at five sampling points, including the early laying stage (HE), three peak laying stages (HPI, HPII, and HPIII), and the late laying stage (HL), with eight randomly selected samples per stage. Shotgun metagenomic sequencing was conducted to characterize the taxonomic structure and functional profiles of the intestinal microbiota and to systematically analyze the diversity and distribution patterns of ARGs. The results showed that most ARGs were harbored by bacteria belonging to the phyla *Pseudomonadota* and *Bacillota*, with *Escherichia coli* serving as the primary carrier of antibiotic resistance genes. Moreover, significant correlations were observed between the co-abundance and co-occurrence of ARGs and MGEs, suggesting that MGEs play a key role in facilitating ARG dissemination. Overall, these findings provide novel insights into the prevalence of ARGs in laying hens across different laying stages and may inform strategies to mitigate the spread of antimicrobial resistance in poultry production systems.

## 1 Introduction

The use of antimicrobials in animal husbandry has significantly contributed to the prevention and control of human and animal diseases. However, excessive use of these chemicals has evolved into a serious health challenge, posing a potential threat to human and animal health, which has garnered widespread concern around the world (1).

The prolonged use of antibiotics in animals not only disrupts the intestinal microecology, but also increases the likelihood of horizontal gene transfer (HGT) of antibiotic resistance genes (ARGs) among microbiota (2). This risk is mainly due to the selection pressure of antibiotics on bacteria, prompting bacteria to undergo genetic changes and thus develop resistance (3). The HGTs between strains of the same or different species can be facilitated by ARG-carrying plasmids, integrons and transposons in bacteria. Even after the drug-resistant strain ceases to exist, the DNA carrying ARGs remains viable in the environment for an extended period due to protection from deoxy nucleotidase (4). This phenomenon increases the risk of transmission of antibiotic resistance and makes it more difficult to prevent and treat bacterial infections. To reduce the emergence and spread of antibiotic resistance and protect human and animal health, the responsible used of antibiotics has become compelling.

Metagenomics offers sufficient sequencing depth to detect the abundance of individual ARGs, making it a powerful tool for anti-microbial resistance (AMR) research (5). Metagenomic analysis can reveal a variety of information such as the type, quantity, gene expression and functional metabolism of gut microbiota and the changes of gut microbiota and resistome at different laying periods. A previous study used metagenomic technology to analyze he circadian oscillations of the microbiome and antibiotic resistance genes in a model of laying hens demonstrated that ARGs are directly affected by microbial β diversity and MGEs and that *E. coli* is an important reservoir of ARGs in feces (6).

Significant research has been conducted on resistance genes of intestinal microbiota in chickens, however investigation of the changes of resistance genes at different laying periods in hens remains insufficient. The special physiological structure of laying hens is that the digestive and reproductive tracts meet in the cloaca, which makes it possible for intestinal microbiota to migrate up the fallopian tube to the funnels (7). This phenomenon undoubtedly poses a potential threat to the health and safety of chicks.

In this study, deep metagenomic sequencing of 40 fecal samples collected at 5 laying periods was performed to construct fecal microbiomes and gene catalogs at different laying periods. The analysis revealed the transfer of ARGs between different bacterial species via MGEs in the gut of laying hens. The study results improved understanding of the transmission mechanism of drug resistance and provide an important scientific basis for improving egg quality and ensuring food safety.

## 2 Materials and methods

### 2.1 Collection of samples

All sampling was carried out at a commercial chicken farm in Qingdao, China, which uses a Type A three-layer cage system, and the living conditions of laying hens are in accordance with the local regulations of living space. No antibiotics were added to the chicken feed. We randomly selected eight chicken cages containing Hyline brown laying hens, and each cage selected one laying hen and wore an anklet number for tracking. Five sampling time points (120, 187, 253, 367, 436 days) with group labels HE, HPI, HPII, HPIII, HL. The sampling time points were as follows: early laying (120 days, daily laying rate <10%, samples labeled HE1-8); peak laying period (187 days, 253 days and 367 days, daily laying rate >90%, samples labeled HPI1-8, HPII1-8 and HPIII1-8); and late laying period (436 days, daily laying rate <80%, sample labeled HL1-8). In commercial layer production, the peak laying period typically lasts for 6–8 months. Although the daily laying rate remains above 90%, physiological and reproductive status, metabolic demands, and endocrine levels can vary during this phase (8). Sampling at multiple time points helps to more accurately capture the dynamic changes in the gut microbiota within the peak laying period.

During the sampling process, strict measures were followed to ensure the purity and integrity of the samples. A clean plastic bag was placed under the cage at 9 am on each sampling day. Then, when our chosen laying hens excreted, their feces naturally fell into the bag. We then carefully removed the plastic bag, quickly transferred the collected feces into a 50 ml sterile centrifuge tube and shipped the sample back to the lab at a low temperature. A total of 40 fecal samples were collected over 5 sampling periods, which were stored at −80 °C until DNA extraction.

### 2.2 DNA extraction, sequencing and data processing

DNA from feces was extracted using the OMEGA Mag-Bind Soil DNA Kit (M5635-02) following the manufacturer's instructions and stored at −20 °C before further assessment. After integrity, purity and concentration tests, qualified DNA was used to construct libraries and sequenced. Each library was sequenced by Illumina NovaSeq platform (Illumina, USA) with PE150 strategy at Personal Biotechnology Co., Ltd. (Shanghai, China).

The DNA sequences were subjected to quality control using FASTP v0.23.2 (9) with the parameters “-q 20 -u 30 -n 5 -y -Y 30 -l 80 –trim_poly_g”. Next, the quality-controlled sequencing data were aligned to the reference genome to remove host genomic information using Bowtie2 v2.4.4 (10). The resulting paired-end sequences were assembled into contigs using MEGAHIT v1.2.9 (11), with the option “–k-list 21,41,61,81,101,121,141′. Next, Contigs longer than 2 kb were binned using MetaBAT2 v2:2.15 (12). Bins with a completeness ≥50%, contamination ≤ 10% and quality score (defined as completeness−5 × contamination) >55 (13) were selected by CheckM2 v.1.2.2 (14). All metagenome-assembled genomes (MAGs) were clustered by dRep v3.4.2 (15) at strains level. The phylogenetic tree generated by GTDB-Tk v2.1.1 (16) and GTDB database (17) was visualized with iTOL (v6, https://itol.embl.de).

### 2.3 Gene prediction and functional analysis

Gene prediction was performed using Prodigal v2.6.1 (18), which only uses contigs longer than 500 bp. The predicted genes were filtered to remove genes shorter than 100 bp. A non-redundant gene catalog was constructed from the predicted genes using MMseqs2 v14-7e284 (19) with the parameter “–cluster-mode 2 –min-seq-id 0.9 –cov-mode 1 -c 0.9” to cluster the genes with the criteria of 90% identity and 90% overlap, resulting in a non-redundant microbial gene catalog comprising 3.1 million genes. Protein-coding genes were compared against the KEGG (20) and CAZy (21) using DIAMOND v2.1.8.162 (22), with parameters “–min-score 60 –query-cover 50”.

### 2.4 ARGs and MGEs prediction

The predicted genes are considered ARGs and MGEs against the CARD (23) (v3.2.6) and MGE (24), with a coverage >80% and identity >80%, using DIAMOND. To assess the abundance of ARGs and MGEs, we aligned the clean reads (20 million reads per samples) against the reference genes using Bowtie2 v2.4.4 with the default parameters, as the search criteria. Using BLASTN v 2.13.0, the ORF annotated to ARGs was compared with the Nucleotide Sequence Database to obtain the bacterial host of ARGs.

### 2.5 Statistical analysis and visualization

Diversity analysis was performed using the “vegan” package. Beta-diversity was assessed via principal coordinate analysis (PCoA) based on Bray-Curtis distance, with PERMANOVA used to test group differences. Statistical tests and models included the Wilcoxon rank sum test to compare community abundance, diversity indices, and functional features, axonomic abundance, and functional features. Mantel tests were conducted using the “LinkET” package. For visualization, heatmaps were generated with “ComplexHeatmap”, Sankey diagrams with “ggsankey”, and gene arrow maps with “gggenes”. Custom charts were created with “ggplo2”, and microbial interaction networks were visualized using “ggraph”. Spearman's correlation analysis was performed to explore associations between gut microbiota, mobilomes, and resistomes.

## 3 Results

### 3.1 Characterization of genomes and genes of samples

A total of 1,015 MAGs were obtained after 99% average nucleotide identity (ANI) dereplication. The genome size of these MAGs ranged from 0.2 Mbp to 5.4 Mbp (average 1.8 Mbp), and the N50 values ranged from 2,808 to 338,700 bp (average 21,527 bp). The GC content averaged 50.2% and ranged from 24.4% to 73.9%. The average completeness was 74.7% and contamination was 2.2%. Among them, 161 genomes (18.7%) met high quality criteria (≥90% integrity and <5% contamination) (Supplementary Table 1).

**Figure 1:**
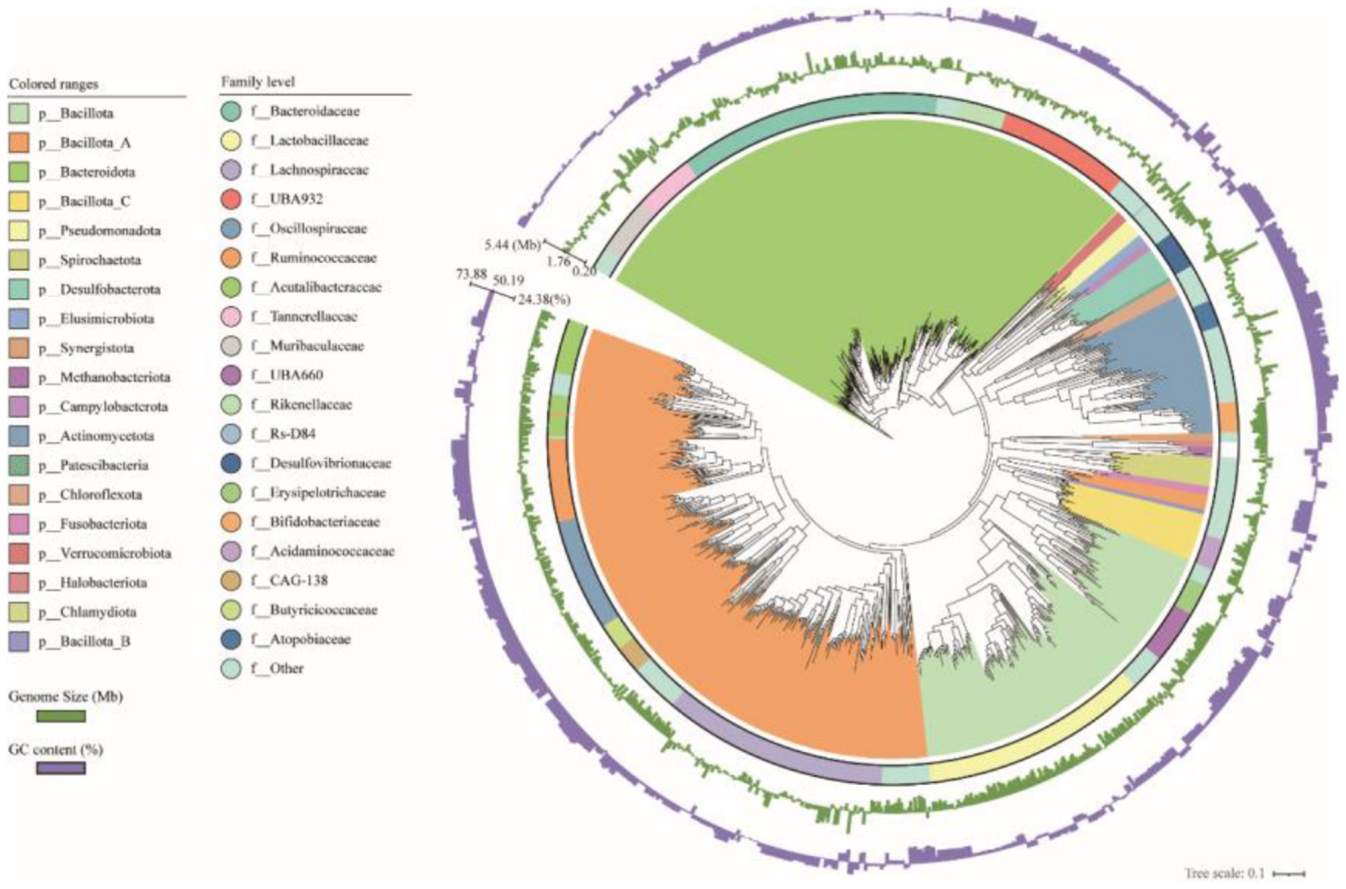
The phylogenetic relationship among 1,007 bacterial genomes. The color coding of each clade corresponds to the phylum level classification of the genomes. The first outer ring shows the classification of genomes at the family level. The second and third rings are bar charts representing the genome size of and GC content of each genome, respectively.

Classification results showed that 1,007 MAGs were bacterial and 8 MAGs were archaeal. The bacterial genomes spanned 17 phyla, 93 families, 229 genera and 401 species. The top three representative bacterial phyla were *Bacillota* (*n* = 535, 53.12%), *Bacteroidota* (*n* = 293, 29.10%) and *Actinomycetota* (*n* = 72, 7.15%). The top three families were *Bacteroidaceae* (*n* = 123, 12.21%), *Lactobacillaceae* (*n* = 108, 10.72%), and *Lachnospiraceae* (*n* = 107, 10.63%), respectively (Figure 1, Supplementary Table 1). Notably, 91 MAGs could not be classified to known species based on the current reference genome database.

After clustering at 90% nucleotide sequence identity, we constructed a non-redundant microbial gene catalog including 3,179,723 genes and an average length of 799.4 bp (length of genes ranged from 102 bp to 22,632 bp). Using BLAST against the NCBI nucleic acid sequence database, a total of 1,189,447 genes of 3,179,723 non-redundant genes could be annotated to bacteria.

### 3.2 Composition of fecal microbiota of laying hens at different laying periods

**Figure 2:**
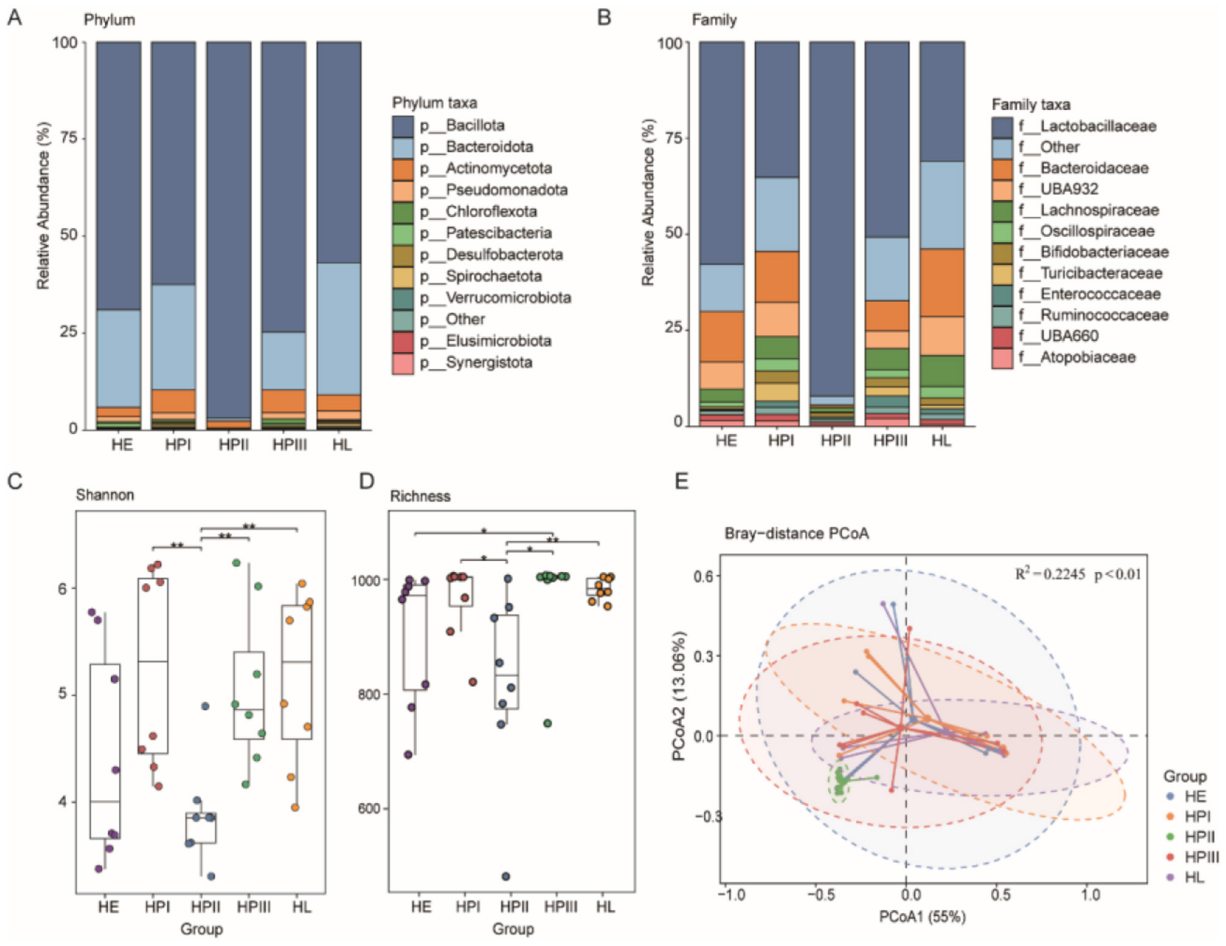
Composition and diversity of intestinal microorganisms in laying hens. **(A, B)** Stacked bar plots display community composition of the gut microbiota at both the phylum and family level, respectively. **(C)** Boxplot showing the Shannon diversity index of intestinal microbiota across different egg-laying stages. Significance levels were determined using the Wilcoxon rank sum test (*p < 0.05, **p < 0.01). **(D)** Boxplot illustrating the species richness of gut microbiota at various egg laying stages. **(E)** Principal Coordinate Analysis (PCoA) plot based on Bray–Curtis distances. Showing β diversity among different egg laying stages. Fecal microbiota function in laying periods.

This study revealed the composition and diversity of the fecal microbiota in laying hens across different production stages by comparing their microbial characteristics. At the phylum level, *Bacteroidota* and *Bacillota* were the dominant bacterial phyla (Figure 2A). At the family level, *Lactobacillaceae* was the most abundant and dominant family (Figure 2B). Except for the HPII group, most of the other groups were similar in the composition of the bacterial spectrum.

We compared the gut microbial diversity across different egg-laying stages. The species richness and diversity during the HPII stage were significantly lower than those in other stages (Figures 2C, D). PCoA analysis based on Bray-Curtis dissimilarity indicated significant differences in microbial community structure between egg-laying stages (*R*^2^ = 0.2245, *p* < 0.01; Figure 2E). At this stage, the energy and hormone fluctuations in laying hens lead to changes in the intestinal environment, which may cause a decline in the abundance and diversity of the microbiota.

**Figure 3:**
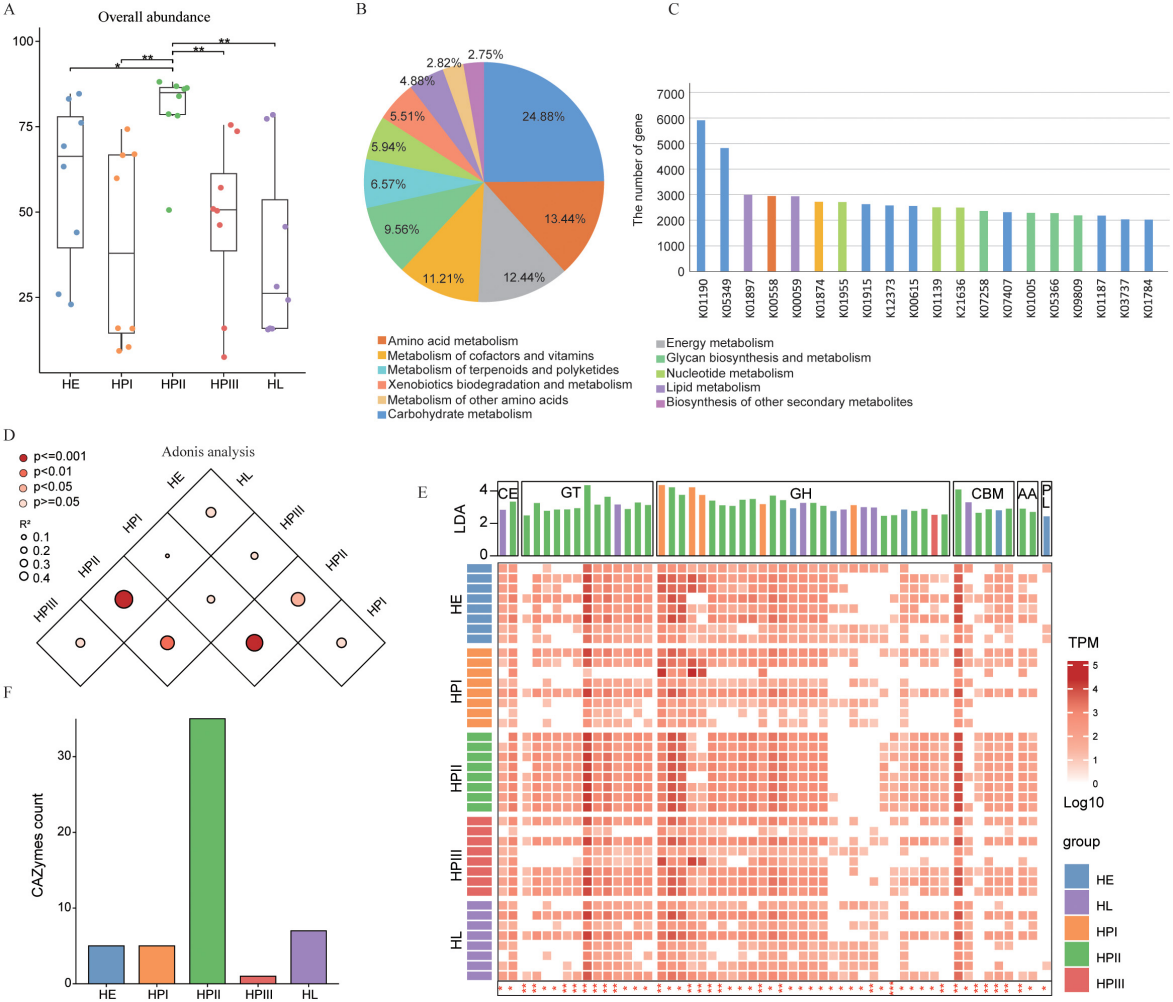
Function and CAZymes distribution of intestinal microorganisms in laying hens. **(A)** The boxplots show the overall abundance of KOs across all groups. Wilcoxon rank sum test: *p < 0.05, **p < 0.01. **(B)** Composition of Biological Processes in Metabolism by KEGG Annotation. **(C)** Number of Genes Annotated to the Top 20 Metabolism-Related KOs. **(D)** Pairnwise PERMANOVA analysis of microbial functions among groups. Darker dots represent smaller *p*-values, whereas larger dots indicate higher R2 values. **(E)** Heatmap showing the relative abundance of CAZymes (log10-transformed RTPM) across all samples. The bar chart above displays LDA scores, highlighting CAzymes with significant group diferences. **(F)** Bar plot summarizing CAZymes significantly enriched in each group.

To investigate the time-dependent variations in microbial functional profiles, we characterized the gene catalog through KEGG Orthology (KO) and CAZymes annotations. A total of 55.62% (1,768,594/3,179,722) of protein-coding genes were assigned to 8,223 KOs. The overall abundance of KOs was significantly higher during the HPII period than other periods (*p* < 0.05, Figure 3A). We selected all metabolism-related KOs and found that Carbohydrate metabolism (*n* = 750, 24.88%) accounted for the largest proportion, followed by Amino acid metabolism (*n* = 405, 13.44%) (Figure 3B). The lacZ gene (K01190) and bglX gene (K05349) encoding beta-galactosidase were the most abundant (Figure 3C). The adonis analysis using Bray-Curtis distance revealed, that the abundance of KOs in HPII group was significantly different from the other groups (*p* < 0.001, Figure 3D).

Subsequently, we studied the distribution of CAZymes in the feces of laying hens at different laying periods. Out of the 3,179,722 predicted proteins, 13.99% (444,866/3,179,722) were found to exhibit at least one CAZymes (Figure 3E, Supplementary Table 2). The most common is glycoside hydrolases (GHs), followed by glycosyltransferases (GTs). LEfSe analysis revealed that gut microbiota of laying hens significantly enriched the highest amount of CAZymes (LDA > 2, *q* < 0.05) at the HPII group (Figure 3F).

### 3.3 ARGs in the fecal microbiota of laying hens

**Figure 4:**
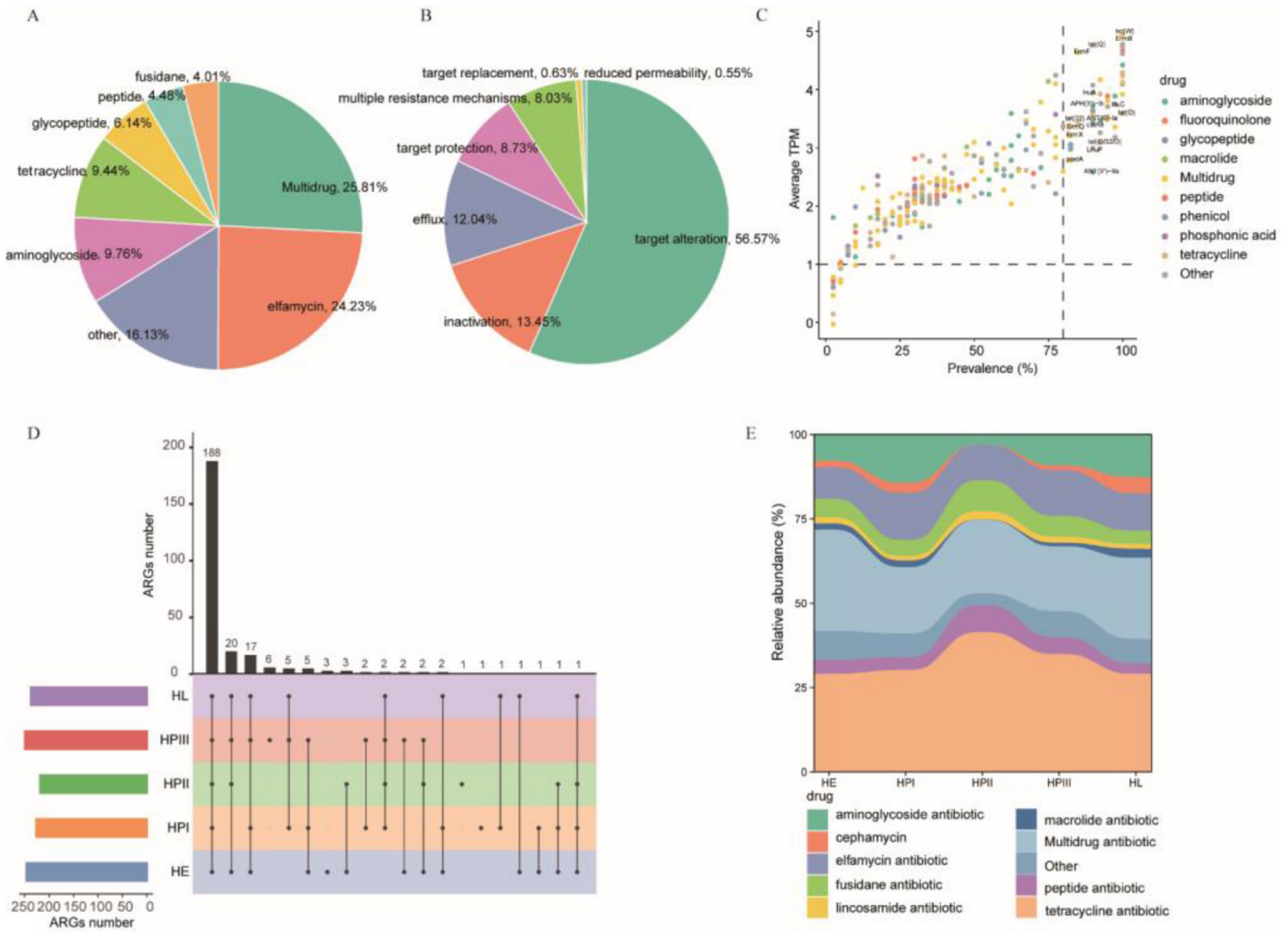
Composition and distribution of ARGs in intestinal microorganisms of laying hens. **(A, B)** The pie charts show the percentage of the predicted ARGs for each drug and the mechanism relative to the total number of predicted ARGs, respectively. **(C)** Prevalence and average relative abundance of the 264 ARGs. **(D)** The upset plot shows shared and unique ARGs across all groups. **(E)** Dynamic changes of antibiotic resistance phenotypes among all groups.

By comparing protein-coding non-redundant genes with the CARD, 1,271 ORFs were identified as representative ARGs. These genes belonged to 273 ARGs (e.g., tet(M), mdtM and ErmB) and 23 antibiotic resistance types (e.g., elfamycin, aminoglycoside and tetracycline) (Supplementary Table 3). Among the 1,271 representative ARGs detected in this study, the most abundant resistance phenotype was multidrug resistance (25.81%), followed by elfamycin antibiotic (24.23%) (Figure 4A). Target alteration was the main resistance mechanism (56.57%), followed by inactivation mechanism (13.45%) and efflux mechanism (12.04%) (Figure 4B).

Subsequently, we assessed the diversity, abundance, variation of ARGs and resistance phenotype at different laying periods. 55 ARGs were found in over 80% of the samples and 18 ARGs were detected in all samples (Supplementary Table 4). The resistance mechanism of these ARGs was antibiotic target protection, antibiotic target alteration, antibiotic inactivation, antibiotic efflux, and multiple resistance mechanisms. The ARGs whose abundance were ranked in the top five were tet(W), ErmB, tet(M), Saur_fusA_FA and tet(Q) (Figure 4C, Supplementary Table 3). Exploring unique and shared ARGs among different hosts enhanced our understanding of the diversity of ARGs. The HPIII group carried the most ARGs, followed by the HE group. The identification of 188 ARGs across the laying periods indicates that these ARGs were widespread among laying hens (Figure 4D). Resistome profiling revealed that tetracycline and multidrug resistance genes were the most prevalent phenotypes across all laying stages, accounting for 29.08% and 19.22% of the total resistance gene abundance, respectively (Figure 4E).

ARGs can confer resistance to host bacteria and potentially contribute to the emergence of multi-drug resistance (MDR) bacteria. Here, the host bacteria harboring these ARGs were identified by taxonomic assignment of overlapping groups containing ARG ORFs. 1,267 representative ARGs were annotated to bacteria, and the other 4 ARGs could not be annotate to any species information (Supplementary Figure 1A, Supplementary Table 3). *Enterobacteriaceae* was the main carriers of ARGs, followed by *Enterococcaceae* and *Staphylococcaceae*. *E. coli* carried 89 ARGs, of which 42 were MDR genes. *E. coli* carried the highest number of ARGs, with the highest number of MDR genes, followed by aminoglycoside (Supplementary Figure 1B).

### 3.4 Exploring the interrelationship among the fecal microbiome, mobilome, and resistome

**Figure 5:**
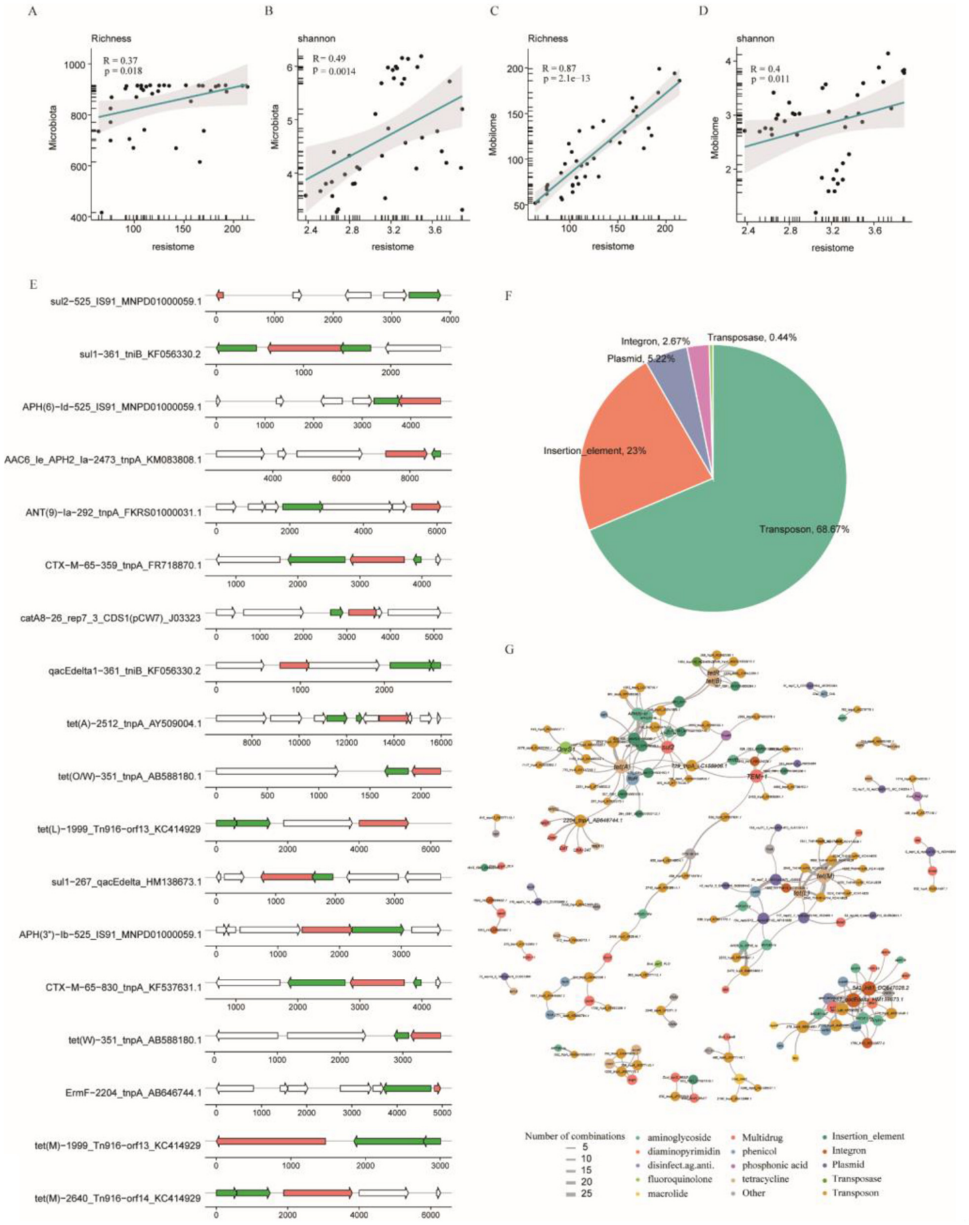
The interrelationship among the fecal microbiome, mobilome and resistome. **(A, B)** Spearman's correlation analyses between the Shannon and richness indices of the gut microbiota and resistome. **(C, D)** Spearman's correlation analyses between the mobilome and resistome based on Shannon and richness indices. **(E)** Arrow diagrams display representative ARG–MGE combinations identified within contigs. **(F)** Pie chart showing the relative proportion of different MGE types. **(G)** Network visualization of ARG–MGE co-occurrence patterns; edge thickness reflects the frequency of detected combinations, while node colors indicate distinct resistance phenotypes or MGE types.

To investigate the impact of gut microbiota composition and temporal dynamics on the genetic determinants of antimicrobial resistance, we integrated microbial community data with resistome profiling to assess their interdependencies. Richness index (*r* = 0.37, *p* = 0.018) and Shannon index (*r* = 0.49, *p* = 0.0014) showed a positive correlation between resistome and microbiome (Figures 5A, B). Specifically, *Bacilli bacterium* was highly correlated with 11 drug resistance phenotypes (*r* > 0.03, *p* < 0.02) (Supplementary Figure 1C, Supplementary Table 5). Together, our data revealed the profound influence of microbial communities on drug-resistant microbiota and the critical role that *Bacilli bacterium* and *Enterococcus* play in this interaction.

MGEs occupy a pivotal position in the horizontal dissemination of ARGs among bacteria. Hence, it is crucial to understand the distribution of these mobile genetic elements in bacteria and their intricate connection with ARGs. By comparing protein-coding genes in the gene catalog to the MGE database established by Pärnänen et al. (24), we identified 900 MGEs in the gene pool (Supplementary Table 6). These MGEs include transposon (68.67%), insertion_element (23%), plasmid (5.22%), integron (2.67%) and transposase (0.44%) (Figure 5F, Supplementary Table 6). Richness index (*r* = 0.87, *p* = 2.1e−13) and Shannon index (*r* = 0.4, *p* = 0.011) showed a positive correlation between resistome and mobilome (Figures 5C, D).

MGEs carrying ARGs are essential facilitators of HGT, driving the evolution of MDR and enabling bacterial populations to adapt to antibiotic-driven selective pressures. Therefore, it is important to study the co-occurrence relationship between MGEs and ARGs. The Tn916 family, specifically Tn916-orf8 and Tn916-orf9, exhibits a tight association with tet(M), which conveys tetracycline resistance, within the same contig (Figure 5E, Supplementary Table 8). The tet(M) was significantly associated with 12 MGEs, including 10 Tn916 genes. To explore potential mobility of resistance genes, we analyzed the co-occurrence of ARGs and MGEs at the contig level. MGEs located within 5 kilobases of ARGs were identified and defined as potential mobile ARG–MGE associations. Our analysis revealed significant co-occurrence patterns between 81 ARGs and 101 MGEs, with 233 distinct ARG-MGE linkage events detected across 382 genomic contigs (Figure 5G, Supplementary Table 7). Genes conferring resistance to aminoglycosides, including aac(6′)-Ie-aph(2′)-Ia, aph(6)-Id, aph(3′)-Ib, ant(9)-Ia, and ant(3′)-IIa, exhibited robust associations with diverse MGEs. Notably, the co-occurrence of aac(6′)-Ie-aph(2′)-Ia and the transposase gene tnpA emerged as the predominant linkage. Furthermore, tetracycline resistance genes were frequently co-located with multiple transposon families, highlighting their potential role in disseminating resistance determinants.

Given that fragmented contigs might not precisely represent the relationships between ARGs and MGEs, we examined their co-abundance patterns and identified strong correlations between 244 ARGs and 255 MGEs based on their abundance (Spearman's correlation: *r* ≥ 0.5, *p* < 0.05, Supplementary Table 8). This indicates that these ARGs are likely horizontally transferred via different MGEs, which could explain their high prevalence and abundance in the samples we tested.

## 4 Discussion

This study conducted metagenomic analysis on 40 fecal samples from laying hens across different egg-laying stages, establishing a comprehensive gene and genome catalog of the fecal microbiota. We identified 3,179,723 non-redundant genes and reconstructed 1,015 microbial genomes. Functional annotation of the gene catalog revealed co-occurrence patterns between ARGs and MGEs, while host-tracking analysis identified bacterial carriers of ARGs.

The gut microbiota was predominantly composed of *Bacillota, Bacteroidota*, and *Actinomycetota*, consistent with previous studies (25, 26). *Bacillota*, the most abundant phylum across all egg-laying stages, is associated with short-chain fatty acid metabolism, particularly propionate and butyrate synthesis (27). During the HPII stage, *Lachnospiraceae* dominated and encoded key carbohydrate-metabolizing enzymes (28, 29) promoting rapid carbon utilization and reducing nutritional niches, which led to a simplified community and decreased microbial diversity.

Abundant bacterial communities are associated with health and production status, while lacking microbial communities are associated with metabolic and physiological disorders (30). Microbial diversity and richness decreased during the HPII group but recovered in subsequent stages. The HPII period showed reduced fecal flora diversity, despite having the highest CAZymes abundance. This phenomenon may be related to changes in host energy metabolism or hormone levels during this stage; however, as these indicators were not directly measured in this study, this interpretation remains speculative and requires further validation. GT and GH were predominant CAZymes, with GT potentially driving temporal microbiota changes (31).

Our results showed that during HPII, there were more tetracycline ARGs [e.g. tet(L) and tet(M)], among which tet(40) increased significantly over these five periods. The tet(40) gene predominantly co-occurs with tnpA, which encodes the core transposase essential for transposon mobility. Through the cut-and-paste transposition mechanism, TnpA mediates the movement of DNA elements within bacterial genomes, thereby facilitating the horizontal transfer of functional genes, including ARGs among different bacterial species (32). Genes encoding resistance to tetracycline were the most common, which may be due to the widespread use of tetracyclines as feed additives and growth promoters (33). Although the farm owner stated that tetracycline had not been used in the feed, it was still detected in the intestinal samples of the laying hens in this study, possibly as a result of tetracycline-induced selection pressure (34). The recent emergence of plasmid-mediated tetX3 and tetX4 in animals and humans has raised public concerns (35), highlighting the need for better surveillance of tetX genes. In addition to participating in nutrient metabolic processes, the chicken gut microbiota also play an important role as a reservoir of ARGs (36).

Our results revealed the changes in the ARGs carried by gut microbiota at different egg-laying periods. *Bacillaceae, Enterococcus* and *Bacilli bacterium* were associated with a variety of drug-resistant phenotypes and may play an important role in promoting the spread of ARGs. In particular, *E. coli* accounted for a large proportion of these ARG-associated contigs, indicating that it plays a key role in harboring ARGs within the gut of laying hens. *Escherichia* and *Enterococcus* were identified as the main hosts of ARG through host tracking analysis (36). Although aminoglycoside antibiotics play an important role in treating bacterial diseases, their high abundance of ARGs may disrupt the healthy balance of the gut microbiota. The enrichment of ARGs may have a negative impact on beneficial microbiota, increasing the proportion of resistant strains and thereby damaging gut health (37).

Through detailed analysis of co-occurrence and co-abundance, we revealed the close interaction between ARGs and MGEs. The repeated co-occurrence of tet(M) with Tn916 family elements (Tn916-orf13/14/15/16) suggests that tet(M) is likely disseminated among different bacterial species via conjugative transfer mediated by the classical conjugative transposon Tn916, thereby promoting the widespread spread of tetracycline resistance (38). Various MGEs further promote the spread of antibiotic resistance between bacteria through the HGT mechanism (39). MGEs are significantly and positively correlated with ARGs in abundance and richness (40). Our study found that the Tn916 family elements (Tn916-orf8/orf9) adjacent to the tetM on the same contig. As MGEs, Tn916 integrates into bacterial genomes and enables conjugative transfer between hosts or intracellular transposition to new genomic loci, contributing to widespread dissemination across commensal and pathogenic bacteria (41). MGEs can horizontally transfer between a diverse range of bacteria, including both pathogens and beneficial human symbionts (42).

It is worth noting that large amounts of ARGs are stored in the intestines of chickens. These chickens may pass on resistance genes to other bacteria through the MGEs-mediated horizontal gene transfer mechanism, which in turn spreads through the food chain to humans (43). In addition, the fecal pathway may also lead to the spread of ARGs in rivers, soil, and other environments, posing a potential risk to human health (44, 45). The distribution pattern and potential exposure risk of airborne ARGs, particularly those in zoonotic pathogens, have been studied in the environments of chicken and dairy farms (46). The baterial α diversity was highest in bioaerosols during peak egg laying, and aminoglycosides were the most abundant ARGs of all egg laying periods (47). Antibiotic-resistant bacteria in layer faces may indeed spread through the environment, and the accelerated emergence and environmental spread of bacteria, gaseous contaminants and ARGs in poultry farm aerosols has become a growing challenge. To effectively address this challenge, it's crucial to strengthen the monitoring of ARGs and MGEs in chicken gut, while developing and implementing scientific livestock waste disposal strategies. These measures will not only support the sustainable development of animal husbandry but will also protect human health and stability of the ecosystem (47).

This study has several limitations: the absence of negative controls or environmental samples during DNA extraction and sequencing, which prevents us from fully excluding the possibility of low-level exogenous DNA contamination. Although all procedures were conducted in a controlled laboratory environment following standard protocols, future work incorporating more rigorous contamination monitoring will help further strengthen the robustness of the findings.

## 5 Conclusion

This study reveals dynamic shifts in gut microbiota composition, function, and antibiotic resistance profiles across laying hen production stages, with the high-production phase (HPII) emerging as a critical transition period marked by reduced microbial diversity and increased ARGs abundance. We identified 1,015 MAGs and 3.18 million non-redundant genes, highlighting *Bacillota* and *Lactobacillaceae* as dominant taxa. Notably, 233 ARG-MGE co-occurrence events were detected, with the Tn916 family specifically associated with tetracycline resistance. These findings underscore the need for phase-specific management strategies to optimize gut health and mitigate resistance spread in poultry production systems.

## Data Availability

The datasets presented in this study can be found in online repositories. The names of the repository/repositories and accession number(s) can be found in the article/Supplementary material.

## Supplementary material

Supplementary Figure 1 Host tracking analysis of ARGs **(A)** Sankey diagram illustrates the relations between taxa (phylum, family, genus, and species) and ARGs, which are categorized by drug class and resistance mechanism. **(B)** A box plot shows the amount of ARGs carried by a particular species in a fecal sample of 40 laying hens. **(C)** Mantel tests were utilized to analyze correlations between microbial species with the resistome.

Supplementary Table 1 Genome information statistics for 1,015 strains.

Supplementary Table 2 LEfSe analysis for CAZymes.

Supplementary Table 3 Characteristic information of the representative ARGs.

Supplementary Table 4 The prevalence of representative ARGs.

Supplementary Table 5 Bacterial taxa are associated with drug phenotypes determined by the Mantel test.

Supplementary Table 6 Annotation results of mobile genetic element genes in open reading frames.

Supplementary Table 7 The statistics of the type and count of ARG-MGE combinations.

Supplementary Table 8 The statistics of the co-abundance relationship between ARGs and MGEs.
