# Supplementary figures and images for: Metagenomics-based characterization of fecal microbiome and resistome of laying hens during the production cycle
Source: Front Vet Sci. 2026 Jan 13;12:1740567. doi: 10.3389/fvets.2025.1740567 (PMC12838260; doi:10.3389/fvets.2025.1740567)

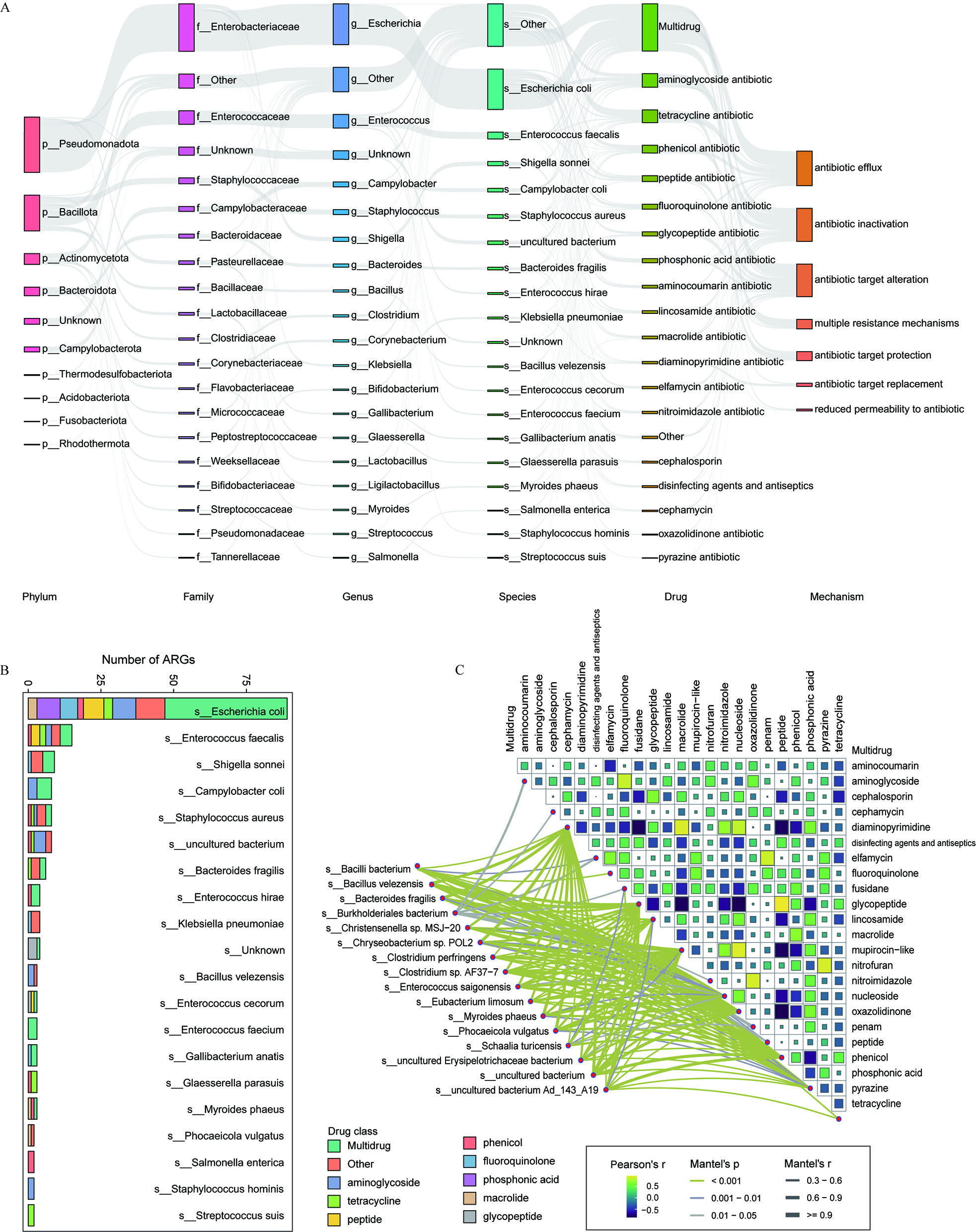

Supplement: Supplementary Figure 1 — Host tracking analysis of ARGs (A) Sankey diagram illustrates the relations between taxa (phylum, family, genus, and species) and ARGs, which are categorized by drug class and resistance mechanism. (B) A box plot shows the amount of ARGs carried by a particular species in a fecal sample of 40 laying hens. (C) Mantel tests were utilized to analyze correlations between microbial species with the resistome. [file Image_1.tif]
